# Comparative analysis of complanadine A total syntheses

**DOI:** 10.3762/bjoc.21.178

**Published:** 2025-10-30

**Authors:** Reem Al-Ahmad, Mingji Dai

**Affiliations:** 1 Department of Chemistry, Emory University, Atlanta, Georgia 30322, United States; 2 Department of Pharmacology and Chemical Biology, School of Medicine, Emory University, Atlanta, Georgia 30322, United States

**Keywords:** biomimetic synthesis, C–H functionalization, complanadine, *lycopodium* alkaloid, skeletal editing, total synthesis, transition metal catalysis

## Abstract

In this review, we summarize and compare four total syntheses of complanadine A, a complex and pseudo-dimeric *lycopodium* alkaloid with promising neurotrophic activity and potential for pain management. These four total syntheses are from the groups of Siegel, Sarpong, Tsukano, and Dai. Each of the four total syntheses contains innovative strategies and creative tactics, reflecting how emerging synthetic capabilities and concepts can positively impact natural product total synthesis.

## Introduction

**Scheme 1:**
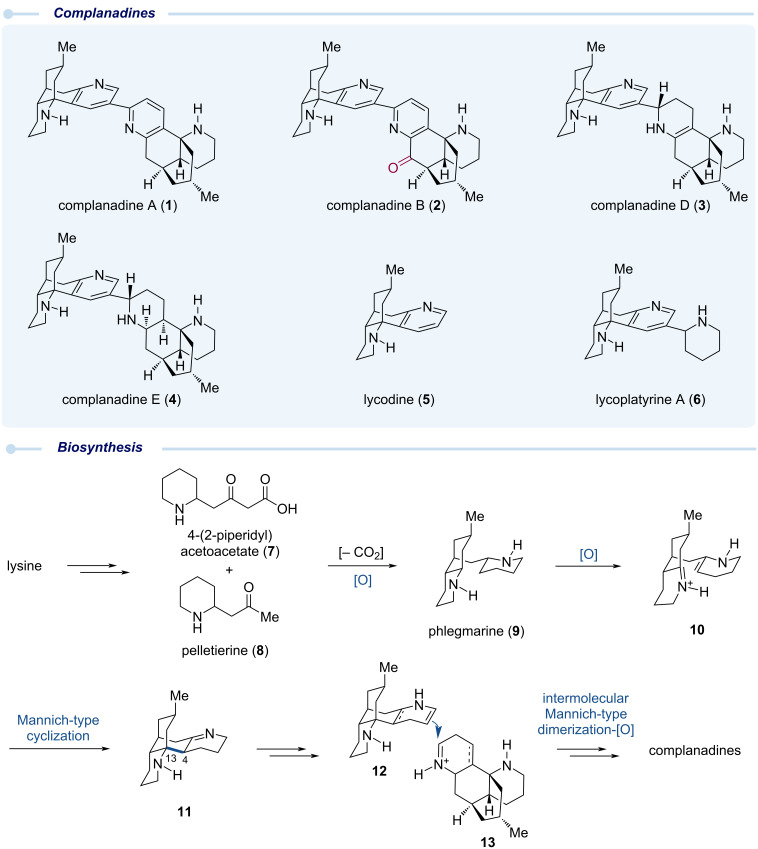
Complanadine natural products and their plausible biosynthesis.

Natural products, owing to their structural complexity, diversity, and therapeutic potential, have continued to serve as inspirations for the development of novel synthetic methodologies and strategies. In turn, these methodological and strategic advancements have significantly improved the efficiency and step-economy of natural product total synthesis. This symbiotic relationship has also helped to accelerate natural product biological evaluation and the subsequent biomedical development [1]. *Lycopodium* alkaloids are one of the largest families of natural products [2], from which famous molecules such as the huperzines have been discovered and advanced into human clinical trials as acetylcholinesterase inhibitors for treating Alzheimer’s disease [3]. Among the *lycopodium* family, complanadine A (**1**, Scheme 1) and its natural congeners such as complanadines B (**2**), D (**3**), and E (**4**) were isolated by Kobayashi and co-workers from the club moss *Lycopodium complanatum* [4–7]. They discovered that complanadine A exhibited neurotrophic activity by enhancing the mRNA expression level for nerve growth factor (NGF) biosynthesis in 1321N1 human astrocytoma cells and NGF production in human glial cells, rendering complanadine A a promising lead compound for neurological disorder treatment. Later, complanadine A was also identified as a lead compound for pain management by Siegel and co-workers [8]. They discovered one of its potential cellular targets as the Mas-related G protein-coupled receptor (GPCR) X2 (MrgprX2), which is highly expressed in neurons and functions as a modulator of pain. Complanadine A serves as a selective agonist of MrgprX2.

Structurally, complanadine A is an unsymmetrical dimer of the tetracyclic lycodine (**5**) via a C2–C3’ linkage [9–10]. Complanadine B is a mono-oxidized analog and complanadines D and E are partially reduced analogs. In addition, natural analogs such as lycoplatyrine A (**6**), isolated as a mixture of diastereomers, were discovered as derivatives of lycodine and an amino acid [11]. Biosynthetically, lysine was proposed to be the starting point of these *lycopodium* alkaloids [12]. Lysine could be advanced to 4-(2-piperidyl)acetoacetate (**7**) and pelletierine (**8**), which would react with each other to deliver phlegmarine (**9**). Double oxidation of **9** would give **10** for a subsequent intramolecular Mannich-type cyclization to forge the C4–C13 bond and produce **11**, which could be further converted to **12** and **13** for an intermolecular Mannich-type dimerization to form the C2–C3’ linkage [13]. Further oxidation state adjustment would give complanadines A, B, D, and E.

Since their isolation, the complanadines, especially complanadine A, have attracted a significant amount of synthetic attention due to their unique structural features and promising therapeutic potential. To date, four total syntheses of complanadine A have been reported from the groups of Siegel [14–15], Sarpong [16], Tsukano [17], and Dai [18], together with one synthetic study from Lewis and co-workers [19]. In this review article, we summarize these four total syntheses, comparatively analyze their strategic novelty and differences, and highlight the impact of enabling methodologies and concepts on the overall efficiency and economy of each total synthesis [20].

## Review

### The Siegel total synthesis – 2010

**Scheme 2:**
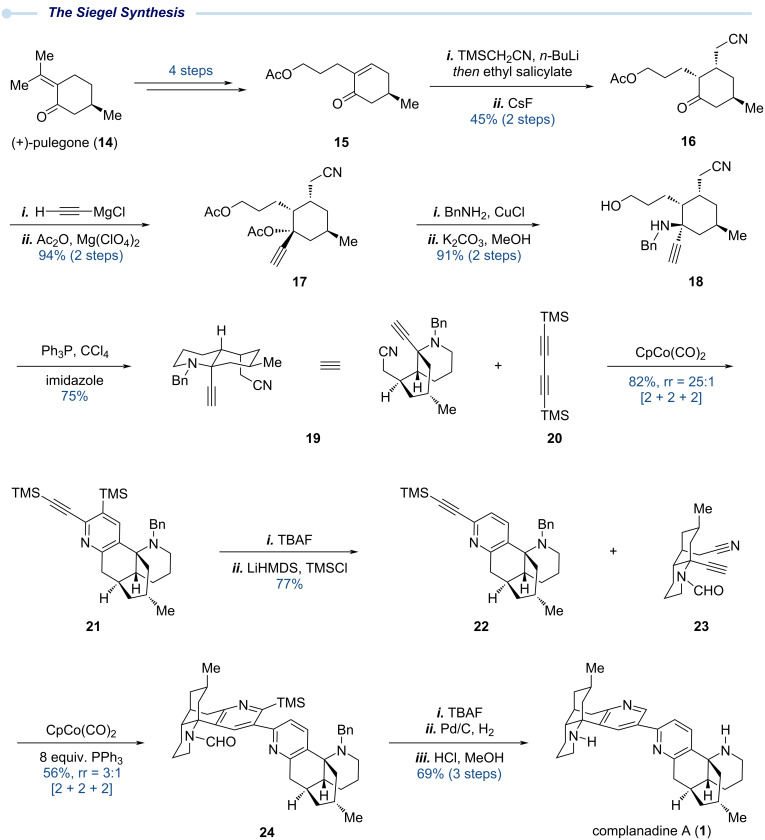
The Siegel total synthesis of complanadine A enabled by [2 + 2 + 2] cycloadditions.

In 2010, Siegel and co-workers reported their total synthesis of complanadine A (Scheme 2). Their synthesis centres on two transition metal-catalyzed alkyne–alkyne–nitrile [2 + 2 + 2] cycloadditions to forge the two pyridine rings encoded by complanadine A [21]. Notably, the C2–C3’ linkage of complanadine A is embedded within the symmetrical bis(trimethylsilyl)butadiyne starting material, elegantly circumventing the challenges associated with the direct construction of this key connection. This strategic maneuver differentiates Siegel’s synthesis from the other three reported approaches. Overall, their synthesis highlights the impact of enabling transition metal catalysis on natural product total synthesis.

The Siegel synthesis starts with chiral pool molecule (+)-pulegone (**14**), which encodes the first stereocenter of the entire sequence. (+)-Pulegone was converted to compound **15** in four steps. Subsequent conjugate addition of the lithium anion of TMS-acetonitrile to **15,** followed by careful one-pot protonation of the resulting enolate with ethyl salicylate and TMS removal with CsF, gave **16** bearing three properly arranged substituents. Grignard 1,2-addition to ketone **16**, followed by acetylation of the resulting propargylic alcohol afforded **17** which was further advanced to **18** via copper-catalyzed selective displacement of the propargyl acetate with benzylamine and hydrolysis of the primary acetate. The primary alcohol of **18** was activated with PPh_3_/CCl_4_, triggering an intramolecular cyclization to afford alkyne–nitrile **19** for the first [2 + 2 + 2] cycloaddition with bis(trimethylsilyl)butadiyne (**20**). This transformation proceeded smoothly under thermal conditions with CpCo(CO)_2_ to afford **21** as the major regioisomer (rr = 25:1) [22]. Removal of the two TMS groups and reinstallation of a single TMS on the alkyne provided pyridyl-alkyne **22** for the second [2 + 2 + 2] cycloaddition reaction which proved nontrivial, with the protecting group on the secondary amine of the alkyne-nitrile moiety and the choice of ligand playing crucial roles. Specifically, when using **19** as the alkyne-nitrile partner, the undesired regioisomer (2,2’-bipyridyl product) was obtained as the major product. Switching the alkyne-nitrile partner from **19,** bearing a benzyl group, to the formyl-substituted partner **23**, combined with addition of 8 equivalents of PPh_3_, successfully inverted the regioselectivity and gave the desired cycloadduct **24** as the major product in 56% yield. From here on, removal of the TMS group with TBAF, followed by hydrogenolytic removal of the benzyl group and acidic hydrolysis of the formyl group, completed Siegel’s total synthesis of complanadine A. In addition, this synthetic route enabled Siegel and co-workers to determine the first biological target of complanadine A. Screening against a panel of cell surface GPCRs revealed that complanadine A acts as a selective agonist of MrgprX2, while the monomer lycodine showed no significant activity towards this target. This finding highlights the importance of complanadine A’s pseudo-dimeric structural motif for its biological function.

### The Sarpong total synthesis – 2010

**Scheme 3:**
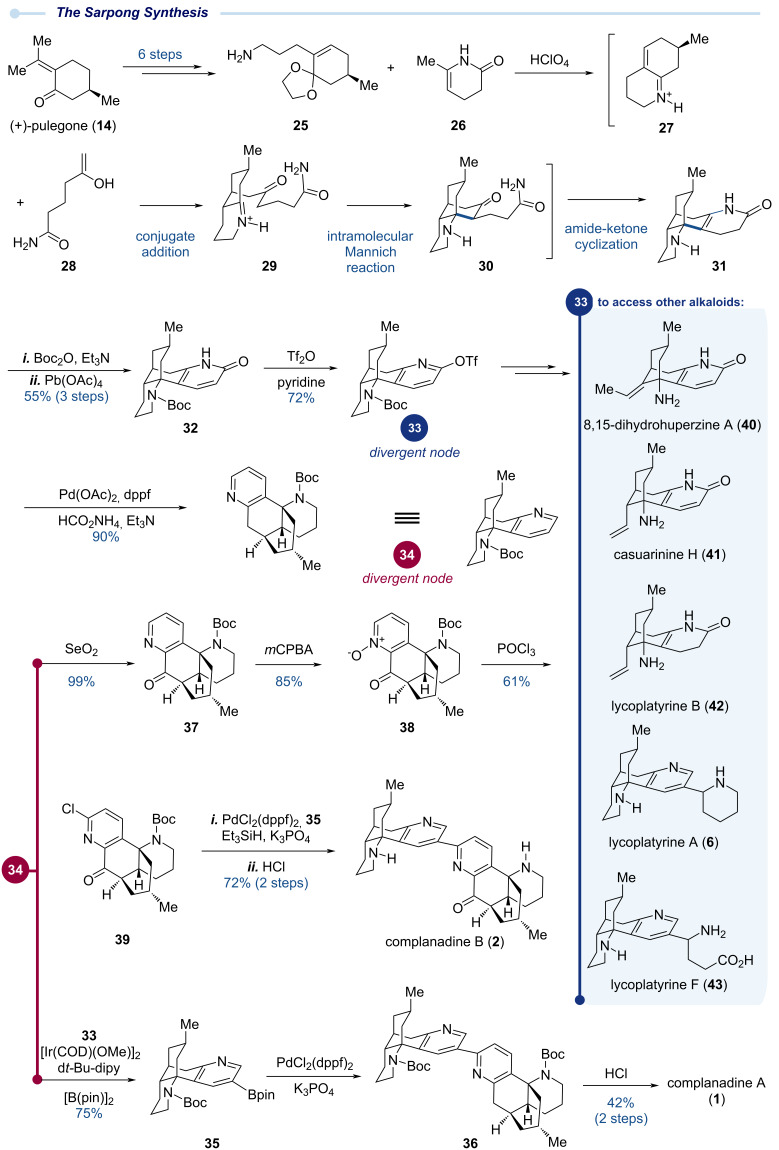
The Sarpong total synthesis of complanadine A enabled by a biomimetic strategy and C–H activation.

In the same year (2010), Sarpong and co-workers reported their total synthesis of complanadine A (Scheme 3). Their synthesis features a biomimetic cascade reaction to rapidly establish the tetracyclic skeleton of complanadine A and an iridium-catalyzed site-selective pyridine C–H borylation followed by a Suzuki–Miyaura cross coupling to forge the C2–C3’ linkage. Their synthesis achieves a high degree of synergy between classic transformations and modern synthetic capabilities, highlighting the importance of biomimetic strategies in total synthesis [23–25].

The Sarpong synthesis utilized (+)-pulegone (**14**) as the starting point as well. They first advanced (+)-pulegone to primary amine **25** with a masked enone moiety, which upon treatment with HClO_4_ underwent ketone release and amine-ketone condensation to form iminium ion **27**. Under the same acidic conditions, enamide **26** underwent hydrolysis and tautomerization to form enol **28**. Conjugate addition of enol **28** to iminium ion **27** gave **29** for the subsequent intramolecular Mannich cyclization to deliver **30**, which continued with an amide-ketone condensation to finally produce **31** in this highly efficient one-step biomimetic cascade sequence. The secondary amine of **31** was selectively protected as a Boc carbamate and the dihydropyridone moiety was oxidized to a pyridone with Pb(OAc)_4_. Pyridone **32** was prepared in 55% from **25** and **26** in three steps. Triflation of the pyridone gave **33** with a triflate at the C2 position for cross coupling to form the C2–C3’ linkage. At this stage, the Sarpong group needed to install a functional group at the C3 position of the pyridine. They creatively solved this challenge with an Ir-catalyzed regioselective C–H borylation developed simultaneously by Ishiyama, Miyaura, Hartwig, and co-workers and Smith and co-workers [26–27]. First, the triflate group of **33** was removed by a Pd-catalyzed reduction with ammonium formate as the reducing reagent. The resulting Boc-protected lycodine **34** underwent Ir-catalyzed C3–H borylation mainly guided by steric factors to provide boronic ester **35** in 75% yield. With the boronic ester handle at the C3 position, the subsequent Suzuki–Miyaura cross coupling between **35** and **33** occurred smoothly to deliver pseudo-dimer **36**, which upon acidic removal of the two Boc protecting groups completed the Sarpong total synthesis of complanadine A.

Efficient access of key coupling intermediates **33** and **34** further enabled Sarpong and co-workers to successfully synthesize other *lycopodium* alkaloids. For example, to synthesize complanadine B with mono-oxidation at one of the two benzylic positions, they started with benzylic oxidation of **34** using SeO_2_ to provide **37**, which was further oxidized to pyridine *N*-oxide **38**. Treatment of **38** with POCl_3_ in DMF delivered 2-chloropyridine **39** for the subsequent Suzuki–Miyaura cross coupling with **35** to form the C2–C3’ linkage. Boc removal then completed their total synthesis of complanadine B [28]. Notably, while complanadine B could be derived from complanadine A via a selective enzymatic oxidation, attempts to achieve this transformation using chemical methods were unsuccessful. For instance, treatment of **36** with SeO_2_ gave a mono-oxidation product at the undesired benzylic position. In addition, from **33** or **35**, Sarpong and co-workers prepared several other *lycopodium* alkaloids including 8,15-dihydrohuperzine A (**40**), casuarinine H (**41**), lycoplatyrines B (**42**), A (**6**), and F (**43**, existing as a mixture of diastereomers) via either a creative “degradation” of the piperidine ring or cross-coupling reactions at the pyridine C3 position [29].

### The Tsukano total synthesis – 2013

**Scheme 4:**
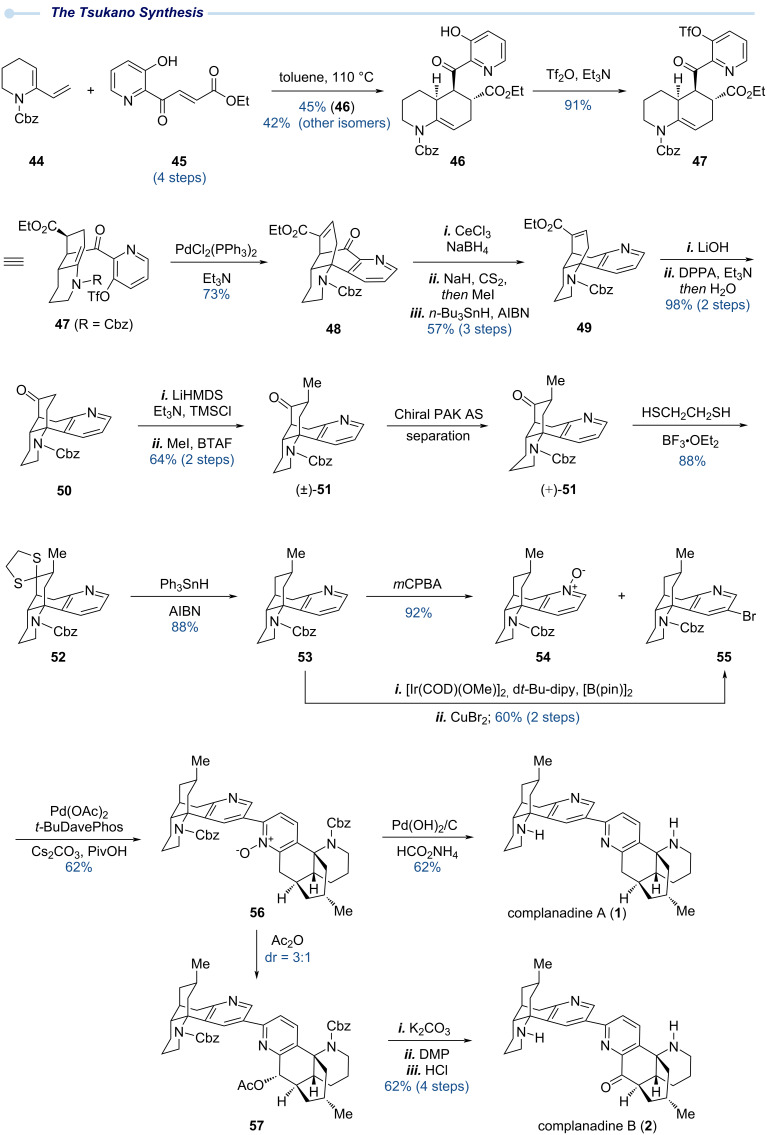
The Tsukano total synthesis of complanadine A enabled by Diels–Alder cycloaddition, Heck cyclization, and directed C–H arylation.

In 2013, Tsukano and co-workers reported their total synthesis of complanadines A and B (Scheme 4). Their synthesis utilizes a Diels–Alder reaction and an intramolecular Heck reaction to build the two six-membered carbocycles embedded in the bicyclo[3.3.1]nonane ring system of complanadine A and a pyridine *N*-oxide directed *ortho* C–H arylation to forge the C2–C3’ linkage.

As shown in Scheme 4, they first prepared diene **44** and dienophile **45** for a thermal Diels–Alder cycloaddition, which afforded a mixture of stereo- (*endo*/*exo*) and regioisomers, among which the desired product **46** was obtained in 45% yield as a racemic mixture. After triflation of the free hydroxy group of **46** to provide **47**, an intramolecular Heck reaction was employed to close the second six-membered carbocycle to deliver **48** in 73% yield. The Diels–Alder reaction and Heck reaction quickly set up the tetracyclic skeleton for subsequent peripheral modifications. First, the ketone functionality of **48** was reduced to a methylene group via a sequence of Luche reduction and Barton–McCombie deoxygenation. The extra ethyl carboxylate was removed via a sequence of LiOH hydrolysis and a Curtius rearrangement using DPPA to form the corresponding acyl azide. Ketone **50** was produced in 98% yield over two steps. The newly formed ketone functionality enabled the introduction of the desired methyl group at its α-position to afford **51** in racemic form. This seemingly straightforward α-methylation turned out to be quite challenging. Tsukano and co-workers eventually utilized a two-step sequence to solve this problem, namely, TMS enol ether formation followed by trapping the enol ether with MeI in presence of benzyltrimethylammonium fluoride (BTAF). At this stage, to prepare optically active natural product, the racemic mixture of **51** was separated using chiral HPLC to afford (+)-**51** and (−)-**51**, which were used to prepare both enantiomers of complanadine A for biological evaluations. With optically active **51** in hand, its extra ketone functionality was reduced via thioacetalization (**51** → **52**) and radical reduction (**52** → **53**) to provide **53**, a diverging point to access C–H arylation partners **54** and **55**. *m*CPBA oxidation of **53** afforded pyridine *N*-oxide **54**. The Ir-catalyzed C–H borylation used in the Sarpong synthesis was again utilized here to introduce a boronic ester at the C3 position, which was further converted to 3-bromopyridine **55** with CuBr_2_. With both **54** and **55**, Tsukano and co-workers employed a remarkable pyridine *N*-oxide directed C–H arylation method developed by Fagnou et al. to forge the C2–C3’ bipyridyl linkage and produce **56** in good yield [30]. From **56**, a one-pot Cbz removal and pyridine *N*-oxide reduction completed their total synthesis of complanadine A. In addition, **56** also served as a key intermediate for their synthesis of complanadine B, which was achieved via a sequence of Boekelheide rearrangement (**56** → **57**), acetate hydrolysis, DMP oxidation and Cbz removal. Based on these results, Tsukano and co-workers suggested that a mono-*N*-oxide intermediate could be involved in the biosynthesis of these dimeric complanadine alkaloids. Importantly, access to both enantiomers of **51** allowed Tsukano and co-workers to prepare both enantiomers of complanadine A. Their further biological evaluation of the complanadines and several synthetic intermediates revealed that the pseudo-dimeric structure, absolute configuration, and oxidation level are important for the observed neurotrophic activity, providing a strong foundation for future analog design and synthesis [31].

### The Dai total synthesis – 2021

**Scheme 5:**
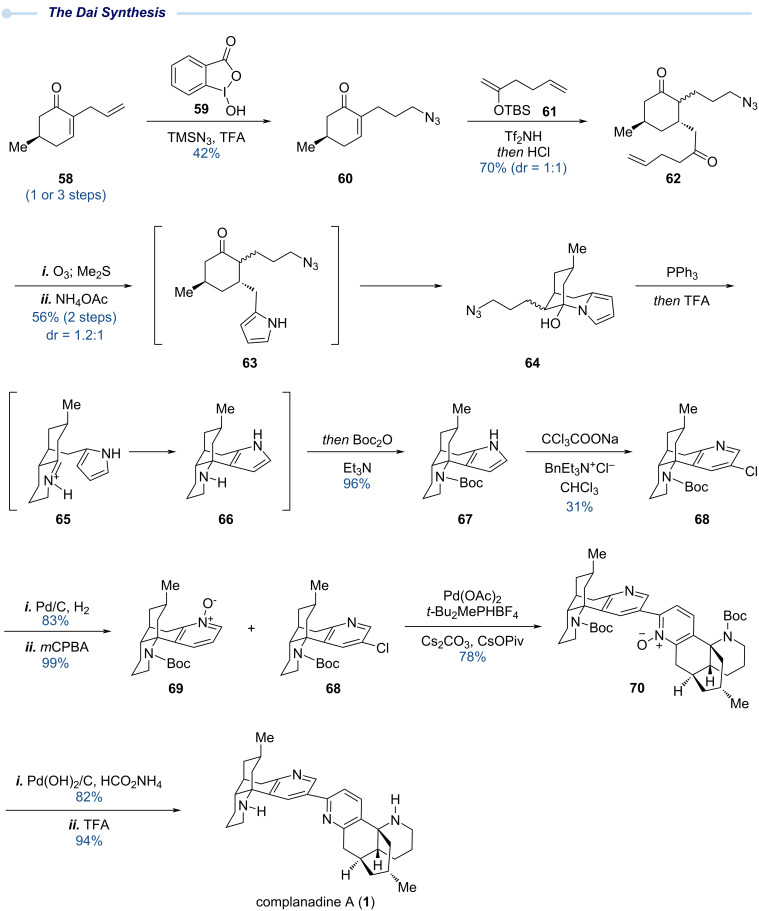
The Dai total synthesis of complanadine A using single-atom skeletal editing.

In 2021, eleven years after the first two total syntheses of complanadine A, Dai and co-workers reported their total synthesis of complanadine A (Scheme 5). Their synthesis features a novel single-atom skeletal editing strategy [32–33] to form the pyridine from a pyrrole and a similar pyridine *N*-oxide directed *ortho* C–H arylation to forge the C2–C3’ linkage as the Tsukano synthesis, but with a much less reactive 3-chloropyridine as the cross-coupling partner.

As shown in Scheme 5, the Dai synthesis starts with compound **58** which can be prepared from (+)-pulegone in three steps or via an organocatalyzed tandem sequence in one step. The terminal olefin of **58** was then converted to a primary azide via an anti-Markovnikov hydroazidation reaction with a combination of **59** and TMSN_3_ recently developed by Xu and co-workers [34]. Mukaiyama conjugate addition between **60** and **61** promoted by Tf_2_NH followed by a one-pot enol ether hydrolysis gave **62** as a mixture of inconsequential stereoisomers. Subsequent oxidative cleavage of the terminal olefin of **62** using ozonolysis followed by Paal–Knorr pyrrole synthesis delivered **63**, which was unstable and spontaneously cyclized to provide **64**. Compound **64** was then advanced to tetracyclic intermediate **67** in a one-pot tandem process, which initiated with Staudinger azide reduction with PPh_3_ to form a primary amine. After reversible hemiaminal opening and amine–ketone condensation, iminium ion **65** was produced for the next pyrrole nucleophilic addition to form a strategically important C–C bond and afford **66**, which was protected as Boc carbamate in the same pot to give **67** in 96% yield from **64**. In this tandem sequence, the nucleophilicity of the electron-rich pyrrole group is essential for the key C–C bond formation. In the next step, the pyrrole group was converted to the pyridine group encoded by the natural product. This single-atom skeletal editing step (**67** → **68**) was achieved using the Ciamician–Dennstedt rearrangement, a reaction discovered back in 1881 [35]. Furthermore, this rearrangement positioned a chloride handle at the desired site for the next C–H arylation. Part of **68** was then converted to pyridine *N*-oxide via Pd/C-catalyzed dechlorination and *m*CPBA oxidation. While a similar C–H arylation strategy was used in the Tsukano synthesis, the 3-chloropyridine **68** used in the Dai synthesis exhibited much lower reactivity compared to the 3-bromopyridine **55** used in the Tsukano synthesis. Thus, a set of new reaction conditions was needed. To solve this reactivity issue, a protocol developed by Stoltz et al. in their jorunnamycin synthesis [Pd(OAc)_2_, *t-*Bu_2_MePHBF_4_, Cs_2_CO_3_, and CsOPiv in toluene at 130 °C] was utilized to afford the C–H arylation in 78% yield with a 1:4 ratio of **68**/**69** [36]. Subsequent reduction of the pyridine *N*-oxide with Pd(OH)_2_/C and H_2_ followed by acidic Boc removal completed their total synthesis of complanadine A (**1**). In addition, 3-chloropyridine **68** enabled Dai and co-workers to prepare simplified analogs of complanadine A for biological evaluation [13].

## Conclusion

**Scheme 6:**
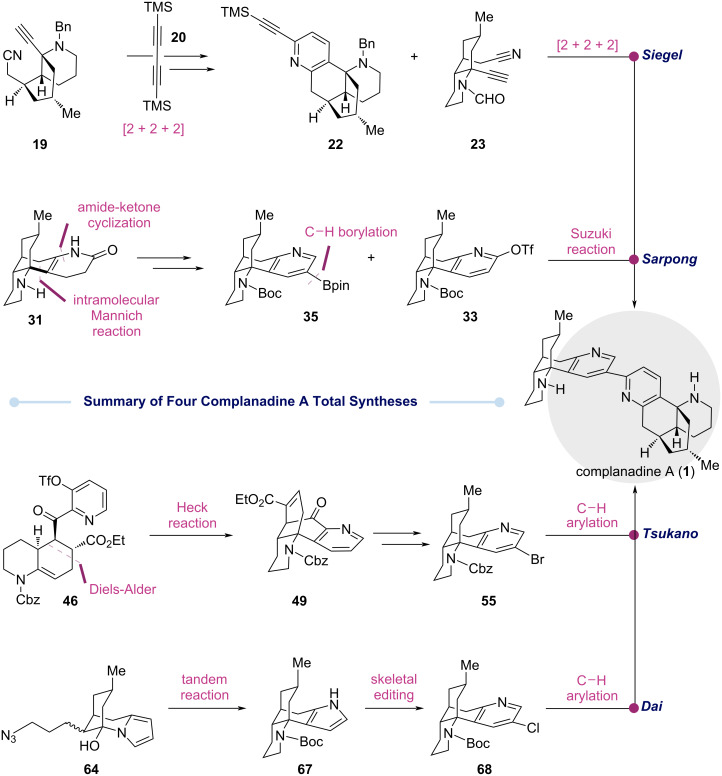
Comparative summary of the four complanadine A total syntheses.

As summarized in Scheme 6, four complanadine A total syntheses were reviewed here. Toward the same target molecule, four strategically unique and different approaches were developed. The Siegel synthesis harnesses the power and efficiency of two Co-mediated [2 + 2 + 2] cycloadditions to build the C2–C3’-bipyridyl moiety encoded by complanadine A. The Sarpong synthesis leverages a biomimetic approach to rapidly assemble the tetracyclic core skeleton and the newly developed C–H borylation to install a boronic ester handle at the desired position for a Suzuki–Miyaura cross coupling to build the C2–C3’ linkage which was hidden in bis(trimethylsilyl)butadiyne (**20**) of the Siegel synthesis. Both the groups of Tsukano and Dai utilized a pyridine *N*-oxide directed C–H arylation to forge the C2–C3’ linkage, but the approaches to prepare the C–H arylation precursors they employed differ completely. In the Tsukano synthesis, a Diels–Alder reaction and an intramolecular Heck reaction were used to build the key ring systems of complanadine A. In the Dai synthesis, they used an electron-rich and nucleophilic pyrrole as the precursor of the electron-deficient pyridine to enable a tandem sequence involving an intramolecular nucleophilic addition of the pyrrole to an iminium ion to form a key C–C bond. The pyrrole group was then converted to the desired pyridine via a single-atom skeletal editing using the 145-year-old Ciamician–Dennstedt rearrangement, completing its long overdue debut in total synthesis. Overall, each of these four total syntheses showcase innovative strategies and creative and enabling tactics including modern transition metal catalysis, C–H activation methods, biomimetic synthesis, classic rearrangements, skeletal editing logic, and others. In addition, these efforts enabled the identification of the potential cellular target of complanadine A, validation of its neurotrophic activity, establishment of preliminary structure activity relationships, and generation of synthetic analogs, all of which pave the way for further study and development of this unique natural product and/or its analogs.

## Data Availability

Data sharing is not applicable as no new data was generated or analyzed in this study.
